# Chemical Discrimination in Turbulent Gas Mixtures with MOX Sensors Validated by Gas Chromatography-Mass Spectrometry

**DOI:** 10.3390/s141019336

**Published:** 2014-10-16

**Authors:** Jordi Fonollosa, Irene Rodríguez-Luján, Marco Trincavelli, Alexander Vergara, Ramón Huerta

**Affiliations:** 1 BioCircuits Institute, University of California San Diego, La Jolla, CA 92093, USA; E-Mails: irenerodriguez@ucsd.edu (I.R.-L.); rhuerta@ucsd.edu (R.H.); 2 AASS Research Center, Örebro University, 70281, Örebro, Sweden; E-Mail: marco.trincavelli@oru.se; 3 Biomolecular Measurement Division, Material Measurement Laboratory, National Institute of Standards and Technology, Gaithersburg, MD 20899-8362, USA; E-Mail: vergara@ucsd.edu

**Keywords:** chemical sensors, open sampling systems, gas turbulence, dynamic chemical mixture, inhibitory support vector machine, gas chromatography

## Abstract

Chemical detection systems based on chemo-resistive sensors usually include a gas chamber to control the sample air flow and to minimize turbulence. However, such a kind of experimental setup does not reproduce the gas concentration fluctuations observed in natural environments and destroys the spatio-temporal information contained in gas plumes. Aiming at reproducing more realistic environments, we utilize a wind tunnel with two independent gas sources that get naturally mixed along a turbulent flow. For the first time, chemo-resistive gas sensors are exposed to dynamic gas mixtures generated with several concentration levels at the sources. Moreover, the ground truth of gas concentrations at the sensor location was estimated by means of gas chromatography-mass spectrometry. We used a support vector machine as a tool to show that chemo-resistive transduction can be utilized to reliably identify chemical components in dynamic turbulent mixtures, as long as sufficient gas concentration coverage is used. We show that in open sampling systems, training the classifiers only on high concentrations of gases produces less effective classification and that it is important to calibrate the classification method with data at low gas concentrations to achieve optimal performance.

## 1. Introduction

The spatio-temporal structure of gas plumes in outdoor environments is mainly determined by turbulent diffusion rather than molecular diffusion. Hence, when a volatile is emitted from its source, the released molecules are carried in the direction of the fluid flow, forming a patchy plume in the downstream direction and decreasing the mean concentration as the volatile molecules spread out. Since, in open environments, air direction and intensity change in time, generated gas plumes have complex, irregular, shifting structures [1,2]. Similarly, when sources of different volatiles are present, the concentration of the compounds change dynamically in time and space, generating non-uniform gas mixtures [3]. In order to reduce the gas concentration fluctuations, and also variations in temperature, humidity and flow, chemical detection systems usually include a measurement chamber or require complex delivery systems to evaluate gas samples. However, sample presentation through a sensing chamber eliminates the important spatio-temporal information contained in the gas plume and does not reproduce the volatile transportation in natural environments: the concentration fluctuations are filtered by the dynamics of the chamber, and the volatiles are blended before the resulting mixture flows through the test chamber.

The design of a chemical detection system that directly samples the environment could provide more information and more realistic representations of the sampled environment, while reducing the size and cost of the complete system. The fluctuations in the gas sensors' signals caused by air turbulence, for instance, can be used to predict the distance to the gas source [4]. Furthermore, architectures based on gas chambers usually require a pumping system, or even human intervention, to bring the sample to the sensors, thereby making the system more complex, expensive, bigger and slower.

The time dependency of gas mixtures generated naturally in open sampling systems makes the typical tasks of identification, quantification or clustering of analytes more challenging. Chemical sensing in open sampling systems has been mainly explored for odor source localization [2]; however, to the best of our knowledge, none of the developed systems considered gas mixtures. Moreover, there is a limited number of studies focused on chemical identification or plume characterization in open environments. Monroy *et al.* proposed a strategy to calibrate a metal oxide (MOX) gas sensor array when exposed to a plume of ethanol [5]. Szczurek and Maciejewska studied the turbulence of an ethanol plume in a 3 × 4.3 × 3.7 *m*^3^ test room [6]. Vergara *et al.* studied the discrimination ability of a sensor array in a wind tunnel exposed to a set of 10 different chemicals [7]. However, in the above-mentioned setups, no mixtures were considered and only one gas was released at a given time. Bennetts *et al.* considered mixtures of ethanol and 2-propanal released at a constant flow in a 5 × 5 × 2 *m*^3^ test room [8]. Using a chemical detection system composed of a metal oxide gas sensor array to identify the compound and a photo ionization detector to quantify the volatile, they obtained a representation of the gas plumes' distribution. In contrast to previous works, we studied the discrimination ability of a standalone sensor array working in a wind tunnel, where two different plumes get mixed naturally in a turbulent environment. Our detection platform contains only sensors based on the same technology (MOX sensors), while the gases were released at different flow rates, thereby generating binary gas mixtures of three volatiles at different concentration levels. Furthermore, by means of a gas chromatography-mass spectrometry (GC-MS) system, a Thermo-Fisher Trace GC ultra coupled with ISQ single quadrupole MS, we were able to obtain an accurate representation of the ground truth and to estimate the mean gas concentration level to which the sensory system was exposed, which, to the extent of our knowledge, was not estimated in any previous setup based on open sampling systems.

In order to evaluate the ability of the sensory system to correctly detect the presence of a compound of interest in a gas mixture, we built classifiers based on inhibitory support vector machines (ISVM) [9]. Nevertheless, we made the collected dataset publicly available for further testing of other machine learning techniques and evaluate their performance to classify naturally mixed gas samples flowing in a turbulent environment (http://archive.ics.uci.edu/ml/datasets/Gas+sensor+array+exposed+to+turbulent+gas+mixtures). We believe that the publication of the dataset can provide a great service to the chemical sensing community and inspire new research in the field of chemical sensing. In the remainder of this manuscript, we first describe the experimental setup utilized in this work, including the chemical detection platform, the wind tunnel and the experimental protocol (Section 2). In Section 3, we present some of the theoretical details on the pattern recognition methodology, followed by the results in Section 4. Finally, we present the concluding remarks drawn from this study in Section 5.

## 2. Experimental Setup

The experimental setup is comprised of a wind tunnel, where two independent gas plumes can be generated, and a detection platform composed of MOX gas sensors. The control of the system is integrated in a computer that commands the signal acquisition from the sensors, the gas flow released at the sources, the wind speed in the tunnel and the operating temperature of the sensors. The experimental setup was adapted from a previous setup developed at the University of California San Diego and described in [7]. In this section, we outline the experiment setup noting the modifications carried out to perform the measurements presented in this manuscript.

### 2.1. Chemical Detection Platform

**Figure 1.**
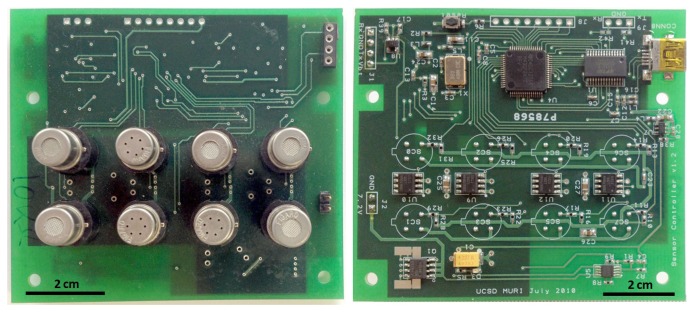
The sensing unit integrates the 8-sensor array along with the signal processing and signal acquisition electronics.

We selected MOX gas sensors to develop our detection platform for their cost-effective design, ease of operation, number of volatiles that can be detected, sensitivity and robustness compared to other technologies [10–12]. Moreover, the correlation between different MOX sensors can be used to extend the lifetime of sensor arrays [13,14] and to correct sensor drift [15,16]. MOX sensors are composed of a metal oxide film, the conductivity of which changes when a reducing/oxidizing gas is present, and an electrically insulated heater. Typically, the heater is placed underneath the sensing layer and is driven by its own electronic circuitry to control the operating temperature of the sensor. The composition of the sensing layer is adapted to show different sensitivities to different analytes: each type of sensing layer responds differently to each chemical stimulus. Hence, we built a detection platform with a diversity of sensor types to obtain a multivariate response to the different gas stimuli. In particular, the sensing unit is composed of 8 off-the-shelf chemical sensors (Figaro USA Inc., Glenview, USA). Table 1 shows the sensors included in the sensing unit, which were specifically selected to detect a broad number of chemicals of special interest. Figure 1 shows the detection platform, including the custom-made electronics to control the sensor operating temperature and to continuously acquire the sensors' signals.

### 2.2. Wind Tunnel Facility

In order to generate two independent gas plumes in an open environment, we modified a 2.5 × 1.2 × 0.4 *m*^3^ wind tunnel facility with two gas sources (labeled as *source1* and *source2*) and a wind flow generator. Each source can be controlled independently to release the selected volatiles at different flow rates, while the wind generator creates a turbulent flow that constantly displaces the introduced volatiles towards the exhaust outlet.

**Figure 2.**
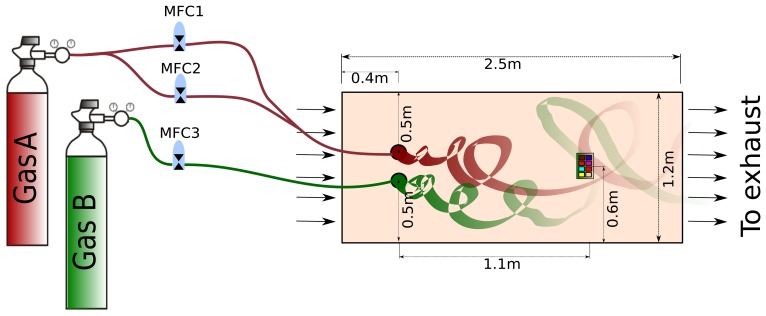
The developed experimental setup can generate two independent plumes. The set of mass flow controllers (MFCs) controls the flow at which the gases are released at each source. The detection platform is exposed to the resulting turbulent gas mixture, while the air carries the emitted molecules towards the exhaust outlet. Two MFCs command the gas flow released at *source1* to increase the maximum flow. The MFC configuration allows accurate adjustment of released flows to make the induced sensors' responses of the same order of magnitude.

The gas concentration of each plume is controlled by a set of mass flow controllers (MFCs). The outputs of two MFCs meet each other before the resulting mixture is released at *source1*, so that the gas from this source can be released at a maximum flow rate of 300 sccm. The output of a third MFC is connected directly to *source2*, which can release gas at a maximum flow rate of 20 sccm. The wind speed is controlled by a multiple-step motor-driven exhaust fan that rotates at a constant frequency (3900 rpm), generating a turbulent flow that can be characterized by the mean speed at the axis of the wind tunnel. Moreover, the wind tunnel is equipped with temperature and humidity sensors (SHT15, Sensirion) to provide accurate measurement of the environmental conditions during the execution of the measurements. Figure 2 shows a diagram of the experimental setup with the exact location of the gas sources and the position of the detection unit. Additional pictures of the wind tunnel are presented in the Supplementary Material of this paper.

### 2.3. Experimental Protocol

Using the experimental setup described above, we exposed the detection unit to mixtures of ethylene with methane or carbon monoxide. The considered volatiles were provided by Airgas Inc. in mixtures of medical dry air, at certified concentrations of 2500 ppm, 1000 ppm and 4000 ppm for ethylene, methane, and carbon monoxide, respectively. The mixtures were created by releasing ethylene at *source1*, whereas the interfering plumes were originated, releasing methane or carbon monoxide, at *source2*. The gases were released at different flow rates. Although the gas concentration at the gas sources was constant for a given volatile, it is important to note that different gas flow rates at the gas sources resulted in plumes of different gas concentrations. Hence, gas sensors showed higher responses to plumes generated with higher flow rates, although the nominal concentration at the gas source was the same for a given volatile.

To generate the experimental dataset, we released each volatile at four different flows (zero, low, medium and high). The complete dataset was composed of 180 measurements, which were performed in a random order. Table 2 shows the number of repetitions performed for each experimental configuration. The total duration of each measurement was 300 s. During the first 60 s, no gas was released at the gas sources. At *t* = 60 s, both sources started to release the corresponding volatile at the specified flow rate. The duration of the gas release was 180 s. Finally, the system acquired the recovery to the baseline for another 60 s. During the whole duration of the experiment, the sensors' signals were acquired constantly every 20 ms, generating 8 time series that were indicative of the gas conditions presented to the sensors.

### 2.4. Gas Chromatography-Mass Spectrometry Analysis

The volatiles utilized to generate the gas plumes were provided in calibrated pressurized gas cylinders (Airgas Inc., Port Allen, LA, USA). Therefore, the concentration level of each volatile was known at the outlet of each source. However, due to the mixing of the volatiles with the air flowing in the wind tunnel, their concentration levels decreased along the wind tunnel. In order to estimate the actual concentration at which the detection unit was exposed, we used a GC-MS system as a gold standard. In particular, we utilized a Trace GC ultra coupled with ISQ single quadrupole MS (Themo Scientific) with a 30 m length, 0.32 mm diameter a GS-GASPRO chromatogram column (Agilent). We used helium as a carrier gas at a flow rate of 1 mL/min. Pictures of the GC-MS system are included in the Supplementary Material of this paper.

The sampling procedure to bring the air sample to the GC-MS system included a pump, a Tedlar bag and a leak-tight syringe for sample injection. Using the mechanical pump (Gilian LFS-113DC) and while releasing the volatiles at the gas sources, we sampled the vicinity of the gas sensor array to fill a RESTEK 7″ × 7″ Tedlar bag. The bag, which had a polypropylene valve with a replaceable septum for further sampling, kept the sample sealed and unaltered. Using a syringe with a built-in push-button valve (Valco Precision Sampling Syringe, Series A-2), 1 mL of the gas sample stored in the Tedlar bag was introduced to the GC-MS for the chromatography analysis. It is important to note that due to the experimental protocol, the obtained mass spectra were the result of the averaged gas sample acquired during the whole sampling time. Hence, the fluctuations in the gas concentration levels due to air turbulence were not expected to be reproduced by means of the GC-MS analysis, but the chromatogram analysis provides a quantification of the number of particles captured during the sampling time.

## 3. Inhibitory Support Vector Machine

In order to test the ability of the gas sensor array to detect ethylene in a turbulent and changing background composed of methane or carbon monoxide on air, we built classifiers based on ISVM classifiers [9]. ISVMs were chosen as classification algorithm given their consistency on two and three class problems, their robustness on small datasets, such as the one used in this work, in which the number of features is significantly larger than the number of patterns, and their successful application to gas classification problems [7,17]. ISVMs are based on the same principles as support vector machines (SVMs) [18], which are arguably one of the most used classification algorithms nowadays, due to their theoretical guarantees and their good performance in a wide variety of domains.

The classifiers are trained on a dataset of *N* samples ${\left\{{\text{x}}_{i}\right\}}_{i=1}^{N}$ in which each point **x***_i_* ∈ ℝ*^M^* is defined by *M* features and belongs to a known class *ŷ_i_* ∈ [1, *L*]_ℕ_. In the context of the gas classification problem considered in this work, each measurement in the wind tunnel corresponds to one pattern for our classification algorithm. The acquired signals from the MOX sensors provide eight time series that follow the gas conditions presented in the wind tunnel during the complete duration of the experiment (300 s). In order to alleviate the computational cost of the classifier, all of the time series were subsampled by selecting the closest measurement in the interval from 3 s to 300 s, in incremental steps of 100 ms. This procedure would be equivalent to sampling the sensors at 10 Hz. We disregarded the first part of the time series to assure the stability of signal acquisition. Hence, the input space of our classification algorithm has 8 x 2961 = 23,688 dimensions (*i.e.*, *M* = 23, 688). Finally, the class of each input pattern is defined by the presence or not of ethylene (*L* = 2): measurements with ethylene were labeled as positive, while experiments without ethylene were assigned to the negative class.

Formally, the objective function is written as follows:

(1) $$\begin{array}{ll} \min_{\text{w}} & E(\text{w})=\frac{1}{2}{\left\|\text{w}\right\|}^{2}+C\overset{N}{\underset{i=1}{\text{∑}}}\overset{L}{\underset{j=1}{\text{∑}}}\eta_{ij}\\ \text{s}.\text{t}. & \eta_{ij}\geq 0\\ & y_{ij}f_{j}(x_{i})-1+\eta_{ij}\geq 0 \end{array}$$

Vector w is formed by the concatenation of the optimal hyperplanes associated with each class; that is, w = (w_1_, w_2_,…, w*_L_*). The cost parameter *C* ∈ ℝ^+^ is responsible for regulating the trade-off between maximizing the generalization capability of the model, represented by the first addend in Equation (1), and maximizing the number of patterns correctly classified, measured by the second term in Equation (1). Specifically, variables {*η_ij_*} are known as slack variables, and their function is to provide room to handle noisy data. The key difference between ISVMs and standard SVMs relies on ISVMs' decision function, which includes an inhibitory term regulated by a scalar parameter *μ*. ISVM's decision function associated with the *j*-th class and the input pattern x*_i_* is defined as follows:

(2) $$f_{j}({\text{x}}_{i})=\langle {\text{w}}_{j},\Phi (x_{i})\rangle -\mu \sum_{k=1}^{L}\langle {\text{w}}_{k},\Phi (x_{i})\rangle$$

Huerta *et al.* showed that the optimal value for *μ* is 1/*L*, which can be directly obtained by minimizing the Lagrangian problem presented in Equation (1) [9]. Finally, the function Φ is a map from the input space to a higher dimensional space where the optimal hyperplanes, w*_i_*, are calculated. Then, it is possible to obtain nonlinear classifiers by using a non-linear function in Φ. The efficiency of SVMs relies on the possibility to formulate their optimization problems in terms of inner products in the feature space and then applying the kernel trick [19]. The kernel trick only requires knowing the inner product between two patterns x and x′ in the feature space, *K* (x, x′) = Φ(x)·Φ(x′), without needing to explicitly compute the image Φ(x) of the patterns, which can be computationally unfeasible due to the high dimensionality of the feature space. In our experiments, we used a radial basis function (RBF) kernel, due to its ability to produce complex decision boundaries. The RBF kernel can be expressed as follows:

(3) $$\text{RBF}(x,x^{\prime })=\exp\left(-\frac{\gamma {\left\|x-x^{\prime }\right\|}^{2}}{M}\right)$$

*M* being the total number of features and *γ* ∈ ℝ^+^ a parameter inversely proportional to the kernel width. Finally, the classification of a data point x̃, *y*(*x̃*), is determined by the maximum of the evaluation functions for each class (Equation (2)); that is,

(4) $$y(\tilde{\text{x}})=\arg\underset{j}{\max}f_{j}(\tilde{\text{x}})$$

## 4. Results

### 4.1. Characterization of the Wind Tunnel

**Figure 3.**
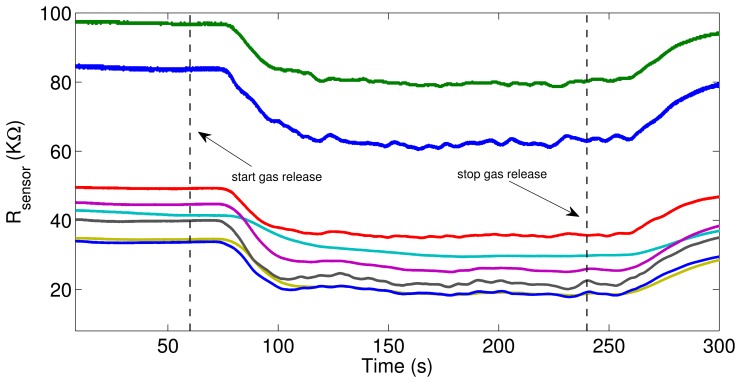
Signals acquired from the sensor unit when being exposed to a dynamic mixture of ethylene and CO. Gas release started at *t*_0_ = 60 s and stopped at *t*_1_ = 240 s. The total duration of the experiment was 300 s.

As previously described, the detection unit provides eight time series that depend on the gas conditions generated in the wind tunnel. Figure 3 shows the acquired signals from the sensor unit when exposed to a dynamic mixture of ethylene and carbon monoxide. The sensors were sensitive to the volatile exposure and gas turbulence; moreover, the observed delays in the sensors' responses (for both decay and recovery phases) with respect to the start/stop gas release were indicative of the time that the released gases needed to travel from the sources to the sensors' position.

In order to quantitatively estimate the airflow and its variability in the wind tunnel, by means of a commercial anemometer (2D Ultrasonic Anemometer, Gill Windsonic), we measured the mean and variance of the wind direction measured at 60 locations. The estimated wind speed at the main axis of the wind tunnel was 0.21 ± 0.005 m/s, confirming the variability of the wind direction and intensity, especially in the center of the wind tunnel, where the walls do not limit the wind direction. For more details on the turbulence in the wind tunnel, the reader is referred to [7].

**Figure 4.**
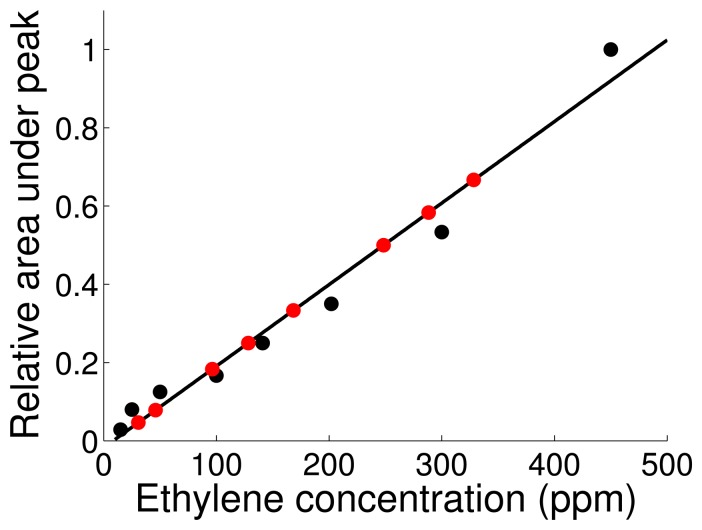
The calibration function (solid line) was adjusted linearly from the calibration points (black markers). Once the calibration function was obtained, it was used to estimate the concentration of ethylene measured in the vicinity of the sensors (red).

The concentration at the gas sources was known, since the concentration in the pressurized gas cylinders was certified by the provider. However, as the gas spread out along the wind tunnel, the gas concentration started to decrease. We calibrated the GC-MS to estimate concentrations of the considered volatiles by means of gas samples with known levels of ethylene. The calibration samples were obtained by mixing, at controlled flows, ethylene at 2500 ppm with air. For each calibration sample, we measured the corresponding area under the peak from the obtained GC-MS spectra. Figure 4 shows the linearly-adjusted calibration function (*r*^2^ = 0.972). The resulting calibration line was utilized to estimate the ethylene concentration of samples taken at the location of the sensory unit. We sampled the air in the vicinity of the sensor array, while generating ethylene plumes at flow rates of 300 sccm, 200 sccm, 140 sccm, 100 sccm, 80 sccm, 20 sccm, 14 sccm and 8 sccm. For each sample, and using the previously obtained calibration curve, we could estimate the amount of the released gas at the source that reached the sensory unit. The measurements corresponding to 20 sccm, 14 sccm and 8 sccm provided direct estimations on the actual ethylene concentration on the sensors' location. However, in order to provide an estimation of the actual concentration of methane and carbon monoxide that reached the sensor array, we used the same ethylene calibration curve to evaluate the fraction of volatiles to which the detection unit was exposed. Finally, we adjusted the fraction of volatiles that reached the gas sensors with the nominal concentration of the corresponding volatiles. The methodology to estimate the ground truth of carbon monoxide and methane showed better accuracy than calibrating the GC-MS system for these gases, since both volatiles showed retention times similar to the retention time of nitrogen. The high concentration of nitrogen in the background air limited the measurement of reliable peak areas for carbon monoxide and methane. Table 3 shows the estimated mean concentrations to which the sensors were exposed.

### 4.2. Gas Classification in Dynamic Mixtures

The aim of the following experiments was two-fold: first, to determine whether it is possible to build a classifier to discriminate ethylene in dynamic mixtures; and second, to analyze the discriminative power of the different concentration levels.

The data were distributed in batches, where each batch represented a different configuration of the mixture of ethylene with the interfering volatile. Each batch was composed of six measurements that were repetitions of the same configuration (see Table 2). The samples within one batch were shuffled to minimize time-dependent effects.

We built binary classifiers to discriminate between the presence and absence of ethylene. The acquired signal from each sensor was normalized with a z-score transform (*μ* = 0, σ = 1). In particular, we built five classifiers: three concentration-specific classifiers trained only with samples at one level of ethylene concentration (*i.e.*, only the batches corresponding to either a low, medium or high concentration of ethylene were used for building the model) and two generic classifiers trained with all of the concentration levels of ethylene. The accuracy of each classifier was estimated from its ability to correctly classify both blank and ethylene samples using test measurements that were not presented during training.

**Figure 5.**
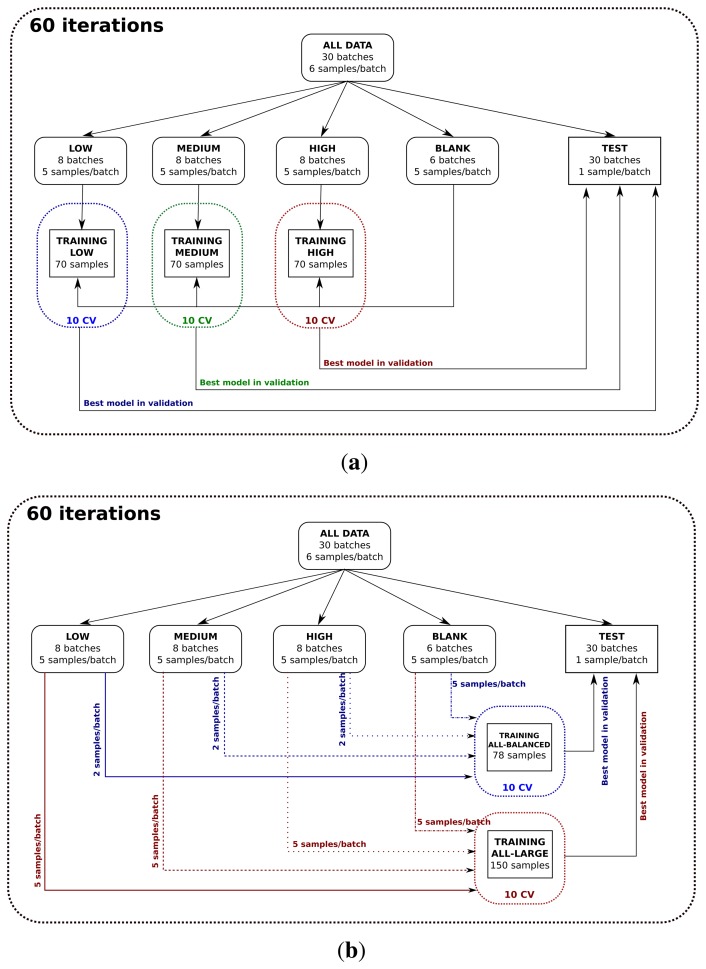
Distribution of the data in training and test datasets for (a) low/medium/high classifiers and (b) classifiers trained with samples at all of the concentration levels (training all-balanced and training all-large).

The methodology to train, validate and test the concentration-specific classifiers is shown in Figure 5a. Let us explain the procedure followed to generate the training, validation and test partitions for the classifier trained with high concentrations of ethylene. This procedure is analogous for the other two concentration-specific models. First, we selected the *n*-th sample from each batch to build the test dataset; hence, the test dataset is composed of 30 samples. Second, we created the training dataset with the remaining 40 samples of high concentration and the 30 samples without ethylene. Third, we trained a binary classifier using the entire training set and selecting the optimal metaparameters (*C* and *γ*) by 10-fold cross-validation. Then, we evaluated the accuracy of the resulting model with the test dataset. We repeated this process six times by setting *n* = 1, 2,…, 6. Finally, we repeated the whole process 10 times by generating different permutations for each batch. The final classification error was computed from the 60 values obtained.

In order to build the classifiers trained with all of the concentration levels, we followed two different methodologies (see Figure 5b). In both cases, the procedure to generate the test set was identical to the one described above, and 10 cross-validations were also used to adjust the metaparameters. However, the samples that conformed to the training set were selected in two different ways. In the first approach, called all-balanced, only two samples from each batch of ethylene were selected to train the classifier. Hence, the size and class prior probabilities of the training sets were similar to those of the concentration-specific classifiers. However, and in order to make use of all of the acquired data, the second strategy (all-large) included all available samples per batch in the training set. Nevertheless, it should be noted that the distribution of classes in this case is significantly different from the distribution used in high/medium/low concentration models, and it is highly unbalanced (120 samples with ethylene *versus* 30 samples without ethylene).

**Figure 6.**
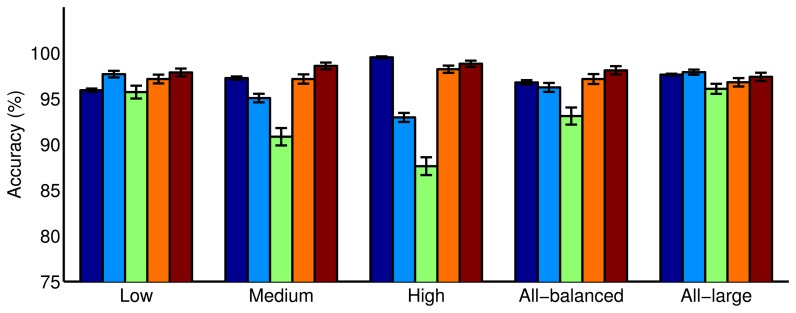
Accuracy of the classifiers when trained under different concentration ranges. Each binary classifier was built with a different training dataset: combining samples without ethylene with samples at either low, medium or high concentration levels (‘Low’, ‘Medium’, ‘High’), with 48 training examples of all ethylene concentrations (‘all-balanced’) and with 150 samples of all ethylene concentrations (‘all-large’). The accuracy in cross-validation is presented (dark blue). Each classifier was tested over the whole test dataset containing samples of all concentrations (light blue) and using only low (green), medium (orange) or high (dark red) ethylene concentration test samples.

The prediction ability of the different classifiers was evaluated using the test sets composed of samples with the same concentration levels used for training, but also with other concentrations, to explore to what extent the models are robust when the system is exposed to conditions not seen during training. Figure 6 shows the accuracy of the classifiers in the cross-validation phase (dark blue) and in testing when using low, medium or high ethylene concentration levels (green, orange and dark red, respectively). In all of the cases, the performance in cross-validation is consistent with the performance of the model when the classifier was trained and tested over the same range of concentrations. This confirms the good generalization properties of the classifiers. Furthermore, we ran a statistical test to figure out the significance of the encountered differences in the performance of the classifiers. In particular, a one-tailed paired *t*-test for equal means, at a significance level of 0.05, was applied. Results are presented in Table S1 (Supplementary Materials), which covers all of the possible comparisons in Figure 6. See the Supplementary Materials for more details on the statistical test.

On the one hand, the results obtained from the statistical test show that models trained with sets of samples that included low concentrations (low, all-balanced, all-large) are statistically significantly better at discriminating low concentrations than other classifiers trained only with medium or high concentrations. Interestingly, no statistical difference when training exclusively at low concentrations or with the large dataset (all-balanced) was found. Although at a different level of significance (compare the *p*-values), the same table also reveals that classifiers trained exclusively with high concentrations are statistically significantly better at classifying medium and high concentrations than a model trained only at low concentrations or with all training data (low and all-large training configurations). The accuracy loss in the classification of samples at high concentrations when training the model only at low concentrations or with all training data is marginal compared to the performance drop in the classification of samples at low concentration when low concentration samples were not presented during training. Hence, we can conclude that the classification of low concentration measurements is more challenging, since all of the models showed the worst accuracy when identifying ethylene at a low concentration. This result is in line with the analysis presented in [17], which reveals the advantage of considering low concentrations in the calibration of gas sensor arrays in order to provide reliable systems. Moreover, models built with low concentrations outperformed the other models when classifying samples at concentration levels different from those used in training. In contrast, models built with medium or high concentrations showed a performance decrease to classify low concentration samples, making their overall classification accuracy lower (Figure 6, light blue). Regarding the generic models trained with all concentrations, the model trained with examples of all concentrations (training all-balanced) also showed low performance when classifying samples of low concentration. This low performance was caused by the relatively small number of training examples of low concentration (16) used to build the model, since when increasing the number of low concentration samples in training (training all-large), the resulting classifier provided the best accuracy of all of the models, reaching a classification accuracy of over 97% in all cases.

**Figure 7.**
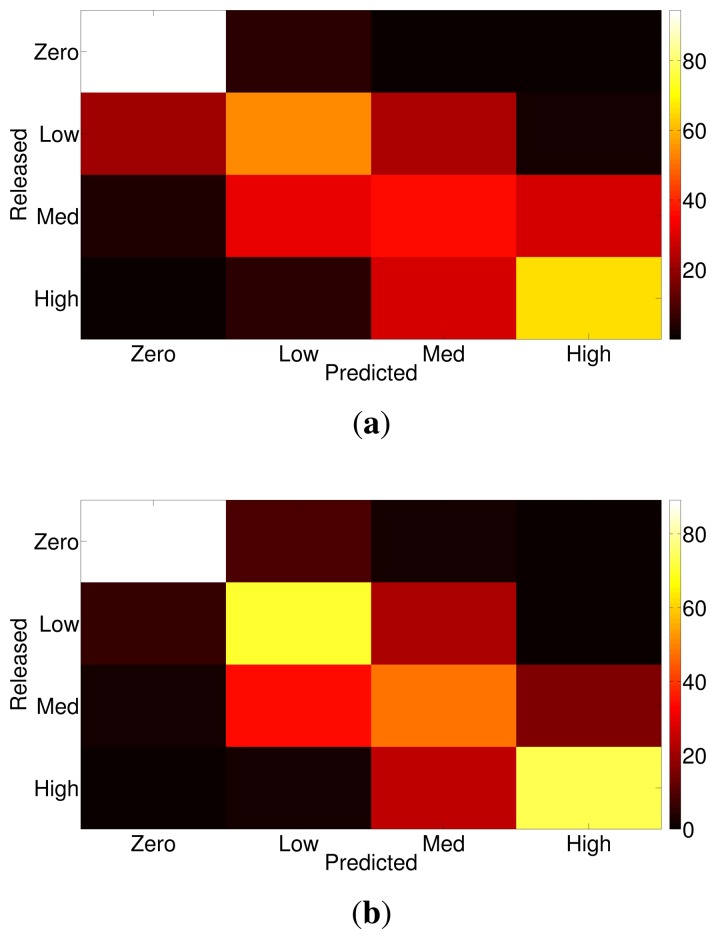
Confusion matrix (in %) to discriminate different ethylene concentration levels. (**a**) Balanced training dataset. (**b**) Using all available samples for training.

We presented the ability of different binary classifiers to identify the presence of ethylene in the provided gas sample, independently of the concentration of ethylene. However, in order to explore the ability of the system to identify the different concentration levels of ethylene present in turbulent gas mixtures, we trained and tested two new classifiers using the four concentration levels of ethylene. Similarly to the methodology for the binary classification, the first classifier was trained with a balanced set of samples (training all-balanced), whereas the second classifier was trained using all of the available training samples (training all-large). Figure 7 shows the confusion matrices for both classifiers, which are able to correctly separate the samples without ethylene from the samples with ethylene. This result is consistent with the binary classifiers, which could identify ethylene with accuracies of over 95% (see Figure 6). However, samples with medium/low concentration levels were mixed by the classifier (see Figure 7). This is in accordance with the estimated concentration level at the sensor unit: low and medium levels correspond to 31 ppm and 46 ppm respectively, which are significantly closer to each other compared to a high concentration (96 ppm) or a zero concentration. Hence, due to the actual concentration levels at the sensors, low and medium concentration levels are more prone to be mixed up by the classifier.

To sum up, the proposed classification methodology allowed an effective discrimination of ethylene in dynamic mixtures and showed the importance of including low concentration measurements in the training dataset, since lower concentration samples are more challenging to classify.

### 4.3. Robustness of the Classifier

We explored the ability of the system to identify ethylene using classification algorithms based on ISVMs due to their consistency on two- and three-class problems, their robustness on small datasets and their proven success in gas classification problems. However, one may be interested in how ISVMs outperform other techniques commonly used for classification of gas sensory data. To benchmark different algorithms, we selected two problems of different complexity. First, we tested the accuracy of the models trained and tested using high concentration levels. Second, models trained with samples at high concentrations were tested on sets of samples acquired at low concentrations. The second test represents a more challenging scenario than the first one.

Using the tools provided in the scikit-learn library [20], we estimated the accuracy of models based on linear discriminant analysis (LDA), K-nearest neighbors, perceptron and SVM. Table 4 shows the mean accuracy of the different classifiers after 60 random partitions of the data. All of the classifiers provide similar accuracy in a simple classification problem. However, the performance of linear models, such as LDA, drops significantly when the task is more challenging. Both standard SVM and ISVM show similar classification accuracy. Hence, the results presented in this paper are robust against the selection of other classification algorithms.

## 5. Conclusions

We exposed a MOX sensor array to dynamic gas mixtures in an open sampling system. The generated gas plumes were mixed naturally along the turbulent flow in the wind tunnel, creating fluctuations in the gas concentration levels. The sensors were exposed directly to the noisy chemical environment, being sensitive to the turbulence present in the system. By means of SVM-based classifiers, we showed that chemo-resistive sensors can successfully identify the presence of a chemical of interest in complex mixtures without inserting a measurement test chamber in the sampling system. A chemical detection system without the requirement of inserting a measurement chamber would be faster, lighter and more versatile. Moreover, it would provide more realistic representation of the gas samples and carry information on the spatio-temporal propagation of the volatiles.

In order to estimate the actual concentration to which the chemical platform was exposed, we used gas chromatography-mass spectrometry analysis. We want to emphasize that although we used GC-MS as a gold standard for the concentration quantification of samples, due to the experimental procedure, such a technique only provides the mean value of the concentration and was not sensitive to the concentration fluctuations and turbulences. Moreover, the cost, size and complexity of the GC-MS system is not convenient for cost-effective and flexible solutions, for which chemo-resistive transduction can be better suited.

Our results show that, in mixture conditions and in a wind tunnel under turbulent flow, high concentration levels are not enough to discriminate low concentrations. It is critical to be able to train the classifier on a low concentration of the gases to achieve optimal performance. This result contrasts with active learning/sampling strategies using controlled gas chambers [17], where higher concentration levels are necessary at the early stages of data sampling, but lower concentrations are need at later stages of the calibration procedure.

We confirmed the robustness in the selection of the classifier with different machine learning techniques commonly used to recover information from gas sensors. However, due to the defined experimental protocol, the detection platform was exposed to the gas stimulus for a fixed period of time. This may not be feasible in other open sampling systems. Algorithms that provide continuous prediction in time may be better suited in applications where an output must be provided while uncontrolled gas conditions change randomly over time.

Finally, we believe that the data acquired from the MOX sensor array exposed to turbulent plumes, along with the estimated ground truth by means of GC-MS system, form a unique dataset that can inspire more research in chemical sensing. Hence, we made the generated dataset publicly available to allow further exploration of dynamic gas mixtures and the test of other classifiers. As claimed by the sensor community [21], it is our belief that sharing experimental datasets will boost chemical detection applications beyond the laboratory.

The generated dataset can be accessed from the UCI repository: http://archive.ics.uci.edu/ml/datasets/Gas+sensor+array+exposed+to+turbulent+gas+mixtures.

## Supplementary Material



## Figures and Tables

**Table 1.** Metal oxide (MOX) sensors included in the sensing unit.

**Sensor type**	**Number of Units**	**Target Gases**
TGS2611	1	Methane
TGS2612	1	Methane, propane, butane
TGS2610	1	Propane
TGS2600	1	Hydrogen, carbon monoxide
TGS2602	2	Ammonia, *H*_2_*S*, volatile organic compounds (VOC)
TGS2620	2	Carbon monoxide, combustible gases, VOC

**Table 2.** Number of samples acquired during the generation of the dataset.

		**Ethylene** @ 2500 ppm			

		20 sccm	14 sccm	8 sccm	0 sccm
CO @4000 ppm	200 sccm	6	6	6	6
	140 sccm	6	6	6	6
	80 sccm	6	6	6	6
	0 sccm	6	6	6	—

Methane @ 1000 ppm	300 sccm	6	6	6	6
	200 sccm	6	6	6	6
	100 sccm	6	6	6	6
	0 sccm	6	6	6	—

**Table 3.** Estimated mean concentration levels at the sensor array.

**Volatile**	**Flow** (sccm)	**Estimated concentration** (ppm)
Ethylene @ 2500 ppm	20	96
	14	46
	8	31

Carbon Monoxide @ 4000 ppm	200	460
	180	397
	140	270

Methane @ 1000 ppm	300	131
	200	115
	100	51

**Table 4.** Accuracy of different classifiers to identify ethylene. The classifiers were trained with high/blank concentration samples and tested with high/blank samples (first column) and with low/blank samples (second column). The mean and the standard deviation (in parenthesis) of the classifier after 60 random repetitions are presented.

	***High/High***	***High/Low***
LDA	98.9 (2.5)	60.2 (9.2)
Perceptron	98.4 (3.2)	81.4 (9.6)
K-NN	94.5 (5.3)	75.6 (9.3)
SVM	99.2 (2.3)	85.2 (8.3)
ISVM	98.8 (0.5)	87.6 (10.8)
