# Supplementary material for: Chemical Discrimination in Turbulent Gas Mixtures with MOX Sensors Validated by Gas Chromatography-Mass Spectrometry
Source: Sensors (Basel). 2014 Oct 16;14(10):19336–53. doi: 10.3390/s141019336 (PMC4239943; doi:10.3390/s141019336)
Supplement: Supplementary file 1 [file sensors-14-19336-s001.pdf]

OPEN ACCESS
sensors
ISSN 1424-8220
www.mdpi.com/journal/sensors

Supplementary Information

Chemical Discrimination in Turbulent Gas Mixtures with MOX Sensors Validated by Gas Chromatography-Mass Spectrometry *Sensors* 2014, *14*, 19336-19353

Jordi Fonollosa [1,*], Irene Rodríguez-Luján [1], Marco Trincavelli [2], Alexander Vergara [3,†] and Ramón Huerta [1]

[1] BioCircuits Institute, University of California San Diego, La Jolla, CA 92093, USA;
E-Mails: irenerodriguez@ucsd.edu (I.R.-L.); rhuerta@ucsd.edu (R.H.)

[2] AASS Research Center, Örebro University; 70281, Örebro, Sweden;
E-Mail: marco.trincavelli@oru.se

[3] Biomolecular Measurement Division, Material Measurement Laboratory, National Institute of Standards and Technology; Gaithersburg, MD 20899-8362, USA;
E-Mail: vergara@ucsd.edu

† Alexander Vergara passed away during the preparation of the manuscript.

* Author to whom correspondence should be addressed; E-Mail: fonollosa@ucsd.edu;
Tel.: +1-858-534-6758.

Figure S1. The flow rates at the gas sources were controlled to generate turbulent plumes along a 2.5 m × 1.2 m × 0.4-m wind tunnel.

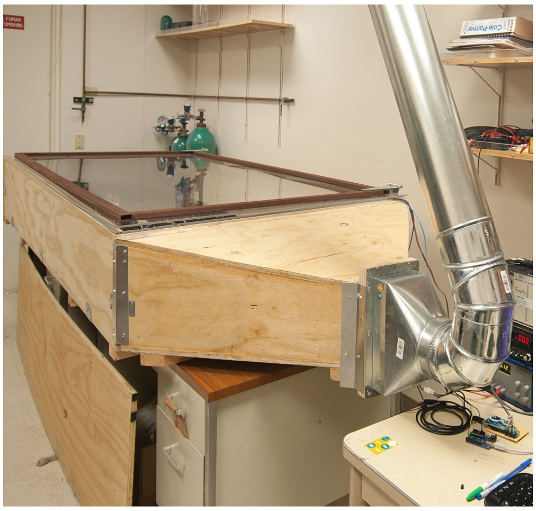

(a)

Figure S1. *Cont.*

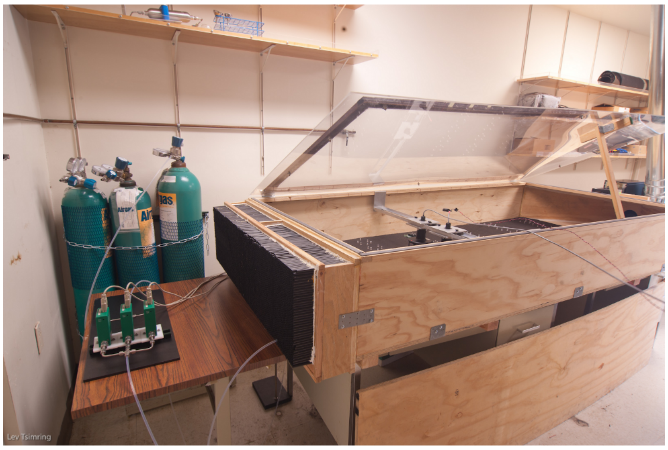

(**b**)

Figure S2. We utilized a Trace GC ultra coupled with ISQ single quadrupole MS (Thermo Scientific) to estimate the ground truth of concentrations.

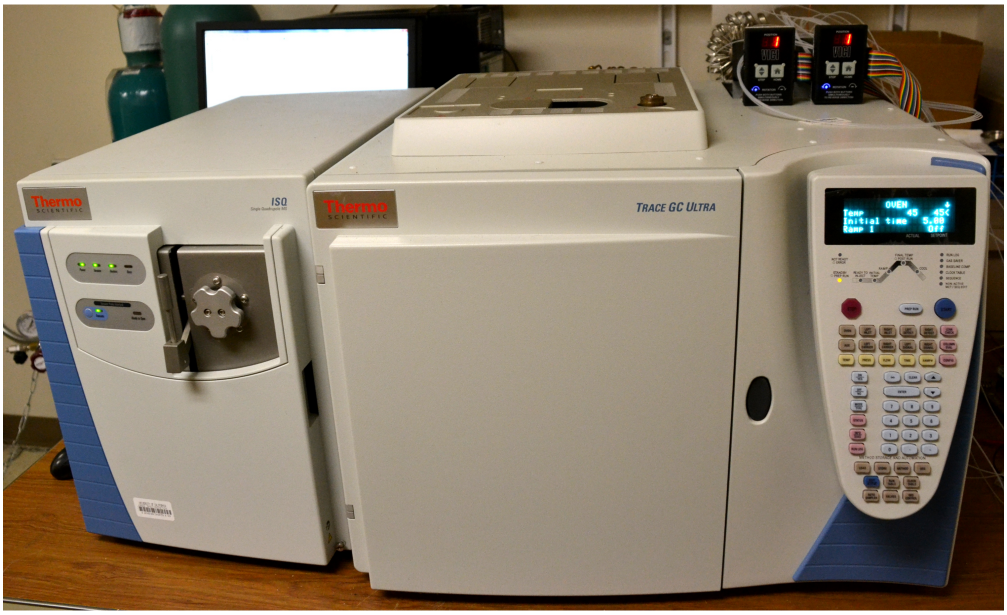

Table S1. One-tailed paired *t*-test. Each row and column in the table corresponds to one of the possible configurations of the training set (low/medium/high concentrations or all-balanced/all-large). The one-tailed paired *t*-test receives as input two sets of error rates, one set corresponding to the error of the model trained with the concentration(s) specified in the row and the other set corresponding to the error of the model trained with the concentration(s) specified in the column. The test is one-tailed because the null hypothesis is that the concentration in the row provides as good or better accuracy than the concentration in the column. The test is paired because both methods were applied to the same 60 data set versions. The column "Test" shows the test set used to evaluate the classification model; L, no ethylene *vs.* low concentration of ethylene (green bar in Figure 6); M, no ethylene *vs.* medium concentration of ethylene (orange bar in Figure 6); H, no ethylene *vs.* high concentration of ethylene (dark red bar in Figure 6); All, no ethylene *vs.* any concentration of ethylene (light blue bar in Figure 6). Results of the test are given in the column labeled "Sig?" by setting the significance level at 5%. Column "*p*-value" shows the *p*-value obtained in each case.

	**Low**			**Medium**			**High**			**All balanced**			**All large**		
	Test	*Sig?*	*p-value*	*Test*	*Sig?*	*p-value*	*Test*	*Sig?*	*p-value*	*Test*	*Sig?*	*p-value*	*Test*	*Sig?*	*p-value*
**Low**				L	Yes	0	L	Yes	0	L	Yes	$1.41 \cdot 10^{-2}$	L	No	$6.55 \cdot 10^{-1}$
				M	No	$5.00 \cdot 10^{-1}$	M	No	$9.54 \cdot 10^{-1}$	M	No	$5.00 \cdot 10^{-1}$	M	No	$2.98 \cdot 10^{-1}$
				H	No	$8.95 \cdot 10^{-1}$	H	No	$9.57 \cdot 10^{-1}$	H	No	$6.50 \cdot 10^{-1}$	H	No	$2.21 \cdot 10^{-1}$
				All	No	$1.55 \cdot 10^{-1}$	All	No	$1.42 \cdot 10^{-1}$	All	No	$2.82 \cdot 10^{-1}$	All	No	$3.27 \cdot 10^{-1}$
**Medium**	L	No	1				L	Yes	$9.80 \cdot 10^{-3}$	L	No	$9.53 \cdot 10^{-1}$	L	No	1
	M	No	$5.00 \cdot 10^{-1}$				M	No	$9.48 \cdot 10^{-1}$	M	No	$5.00 \cdot 10^{-1}$	M	No	$3.03 \cdot 10^{-1}$
	H	No	$1.05 \cdot 10^{-1}$				H	No	$6.80 \cdot 10^{-1}$	H	No	$2.07 \cdot 10^{-1}$	H	Yes	$2.16 \cdot 10^{-2}$
	All	No	$8.45 \cdot 10^{-1}$				All	No	$5.66 \cdot 10^{-1}$	All	No	$6.49 \cdot 10^{-1}$	All	No	$7.09 \cdot 10^{-1}$
**High**	L	No	1	L	No	$9.90 \cdot 10^{-1}$				L	No	1	L	No	1
	M	Yes	$4.61 \cdot 10^{-2}$	M	No	$5.19 \cdot 10^{-2}$				M	No	$5.75 \cdot 10^{-2}$	M	Yes	$1.08 \cdot 10^{-2}$
	H	Yes	$4.27 \cdot 10^{-2}$	H	No	$3.20 \cdot 10^{-1}$				H	No	$1.04 \cdot 10^{-1}$	H	Yes	$6.50 \cdot 10^{-3}$
	All	No	$8.58 \cdot 10^{-1}$	All	No	$4.34 \cdot 10^{-1}$				All	No	$6.19 \cdot 10^{-1}$	All	No	$6.93 \cdot 10^{-1}$
**All balanced**	L	No	$9.86 \cdot 10^{-1}$	L	Yes	$4.67 \cdot 10^{-2}$	L	Yes	0				L	No	$9.96 \cdot 10^{-1}$
	M	No	$5.00 \cdot 10^{-1}$	M	No	$5.00 \cdot 10^{-1}$	M	No	$9.42 \cdot 10^{-1}$				M	No	$3.09 \cdot 10^{-1}$
	H	No	$3.50 \cdot 10^{-1}$	H	No	$7.93 \cdot 10^{-1}$	H	No	$8.96 \cdot 10^{-1}$				H	No	$1.30 \cdot 10^{-1}$
	All	No	$7.18 \cdot 10^{-1}$	All	No	$3.51 \cdot 10^{-1}$	All	No	$3.81 \cdot 10^{-1}$				All	No	$5.57 \cdot 10^{-1}$
**All large**	L	No	$3.45 \cdot 10^{-1}$	L	Yes	0	L	Yes	0	L	Yes	$3.70 \cdot 10^{-3}$			
	M	No	$7.03 \cdot 10^{-1}$	M	No	$6.97 \cdot 10^{-1}$	M	No	$9.89 \cdot 10^{-1}$	M	No	$6.92 \cdot 10^{-1}$			
	H	No	$7.79 \cdot 10^{-1}$	H	No	$9.78 \cdot 10^{-1}$	H	No	$9.94 \cdot 10^{-1}$	H	No	$8.70 \cdot 10^{-1}$			
	All	No	$6.73 \cdot 10^{-1}$	All	No	$2.91 \cdot 10^{-1}$	All	No	$3.07 \cdot 10^{-1}$	All	No	$4.43 \cdot 10^{-1}$			

© 2014 by the authors; licensee MDPI, Basel, Switzerland. This article is an open access article distributed under the terms and conditions of the Creative Commons Attribution license (http://creativecommons.org/licenses/by/4.0/).
